# Glomerular cell cross talk in diabetic kidney diseases

**DOI:** 10.1111/1753-0407.13304

**Published:** 2022-08-23

**Authors:** Ruixue Dong, Youhua Xu

**Affiliations:** ^1^ Faculty of Pharmacy Macau University of Science and Technology Taipa Macau People's Republic of China; ^2^ Faculty of Chinese Medicine Macau University of Science and Technology Taipa Macau People's Republic of China; ^3^ Department of Endocrinology Zhuhai Hospital of Integrated Traditional Chinese and Western Medicine Zhuhai People's Republic of China

**Keywords:** cell cross talk, diabetic kidney disease, glomerulus, 糖尿病肾病, 细胞相互作用, 肾小球

## Abstract

Diabetic kidney disease (DKD) is a severe microvascular complication of diabetes mellitus. It is the leading inducement of end‐stage renal disease (ESRD), and its global incidence has been increasing at an alarming rate. The strict control of blood pressure and blood glucose can delay the progression of DKD, but intensive treatment is challenging to maintain. Studies to date have failed to find a complete cure. The glomerulus's alterations and injuries play a pivotal role in the initiation and development of DKD. A wealth of data indicates that the interdependent relationship between resident cells in the glomerulus will provide clues to the mechanism of DKD and new ways for therapeutic intervention. This review summarizes the significant findings of glomerular cell cross talk in DKD, focusing on cellular signaling pathways, regulators, and potential novel avenues for treating progressive DKD.

## Graphical abstract

**Highlights**

- Glomerular cell cross talk is essential for maintaining the glomerular filtration barrier.
- In‐depth exploration of intraglomerular signaling pathway changes during diabetic kidney disease may help discover new therapeutic targets.

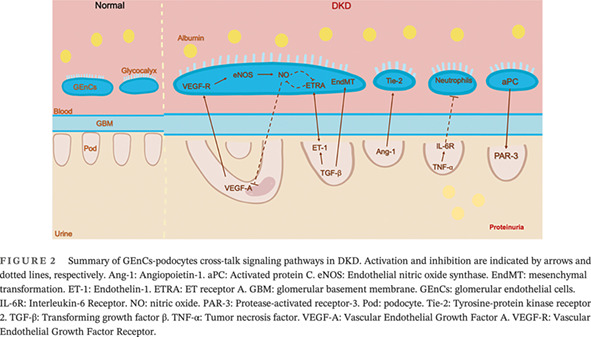

## 摘要

糖尿病肾病(DKD)是糖尿病(DM)的一种严重微血管并发症。它是终末期肾病(ESRD)的主要诱因, 其全球发病率一直在以惊人的速度增加。严格控制血压和血糖可以延缓DKD的进展, 但强化治疗很难维持。到目前为止, 还没有找到完全治愈的方法。肾小球的改变和损伤在DKD 的发生和发展中起着关键作用。大量数据表明, 肾小球内驻留细胞之间的相互作用关系将为DKD的发病机制提供线索, 为治疗干预提供新的途径。本文综述了肾小球细胞相互作用在DKD中的重要研究结果, 重点介绍了对细胞信号通路、调节因子和DKD潜在新途径的研究进展。

## 1 INTRODUCTION

The function of the mammalian kidney is to filter blood and concentrate metabolic waste into the urine. This is critical for fluid homeostasis and osmotic pressure regulation, which occurs primarily in complex structures called the renal glomerulus.^1^ The glomerulus can retain the valuable macromolecular components in the plasma so that the excreted urine contains only traces of proteins.^2^ Diabetic kidney disease (DKD) is a highly prevalent complication of diabetes mellitus, influenced by both genetic and environmental factors.^3^ The clinical manifestation of DKD is persistent proteinuria, which is a manifestation of a compromised glomerular filtration barrier (GFB).^4^ As the disease progresses, kidney function declines and eventually develops into end‐stage renal disease (ESRD) and even premature mortality.^5^

According to the International Diabetes Federation (IDF), in 2021, about 536.6 million people worldwide had diabetes, a figure likely to increase to 783.2 million by 2024.^6^ In the past, the clinical treatment of DKD was limited to blood glucose control and renin‐angiotensin system (RAS) blockade. Morbidity and mortality remain unchanged high.^7^ Encouragingly, sodium glucose cotransporter 2 (SGLT2) inhibitors are a new class of glucose‐lowering agents recently approved in type 2 diabetes mellitus (T2DM).^8^ The mechanism of action of these drugs is to enhance urinary glucose excretion by early inhibition of glucose reabsorption in the proximal renal tubules.^9^ In addition, a new generation of nonsteroidal selective mineralocorticoid receptor (MR) antagonists has also appeared in indications for DKD, of which finerenone significantly reduced proteinuria in a short‐term trial of both chronic kidney disease (CKD) and T2DM patients.^10^ Although the emergence of these two drugs brings new possibilities for treating DKD, there are still many challenges to fully applying them in clinical practice, such as relatively high prices and unclear long‐term side effects. Hence, a better understanding of the mechanism of the development of DKD is fundamental to finding better treatments. Recent research has shown that to maintain the filtration function, cross‐communication between cells in the glomerulus must occur,^11^ and the glomerulus is the target of injury and ultimate scarring in a wide variety of kidneys diseases.^12^ This suggests that a thorough understanding of the molecular signaling mechanisms between intraglomerular cells could help identify potential therapeutic options for DKD. Here, we will highlight recent findings of cell cross talk, which regulates glomerular barrier function in DKD conditions.

### 1.1 Structure and function of the glomerular

**FIGURE 1:**
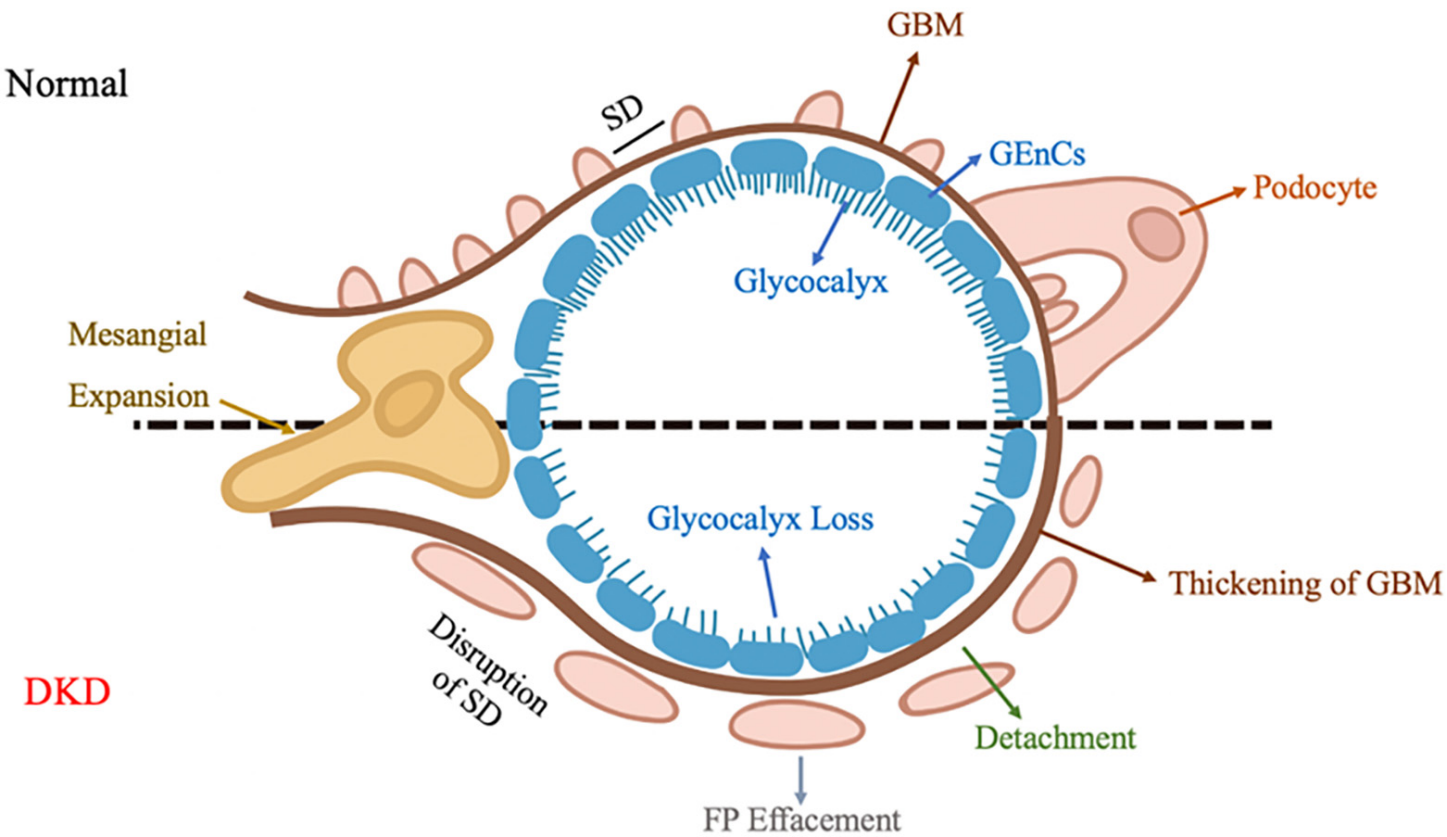
Characteristic glomerular changes of DKD. DKD: diabetic kidney disease. FP: foot processes. GBM: glomerular basement membrane. GEnCs: glomerular endothelial cells. SD: slit diaphragm.

The glomerular capillary barrier is one of the most complex biofilms to date， with a highly specialized structure that restricts the passage of large molecules and serum albumin into Bowman's space, but it is still highly permeable to small molecules and water.^13^, ^14^ The mature glomerulus consists mainly of mesangial cells, podocytes, parietal epithelial cells (PECs), and glomerular endothelial cells (GEnCs).^2^ These four types of cells interact and are closely intertwined through complex cell–cell biological processes and harmoniously differentiate to achieve glomerular structure and function integrity.^15^ Mesangial cells, also known as mesenchymal cells, are located between the glomerular capillary loops and together with the extracellular matrix (ECM) form the mesangium. The primary function of mesangial cells is to maintain the structural stability of the glomerular vasculature and modulate capillary blood flow.^16^ Under pathological conditions, mesangial cells proliferate excessively, and ECM deposition increases.^17^ Podocytes are terminally differentiated epithelial cells with detailed projections called foot processes (FPs), and there are slit diaphragm (SD) proteins between FPs, which allow for podocyte‐to‐podocyte contact and form a size selectivity barrier for the passage of molecules and blood filtration.^18^, ^19^ In DKD patients with proteinuria and glomerulosclerosis, podocyte FP disappearance, hypertrophy, properties decrease, and even detachment from glomerular basement membrane (GBM) or death can occur.^20^ PECs are located on the internal surface of the Bowman's capsule and can self‐renew and be induced to transdifferentiate to other cells. Under specific pathological conditions, PECs can differentiate toward podocytes to complement the reduced number of podocytes and play a potential role in glomerular repair.^21^ GEnCs are highly specialized cells covered with the glycocalyx layer. The high charge of the glycocalyx can selectively restrict the passage of negatively charged molecules, such as albumin, and limit the adhesion of leukocytes and platelets to endothelial cells, thereby alleviating inflammation and thrombosis; glycocalyx is reduced in DKD.^22^, ^23^ Because of direct contact with the blood, lesions in GEnCs can be detected before the onset of proteinuria.^24^, ^25^ GEnCs sit on the GBM and opposite the podocytes, forming an interconnected GFB.^26^ GBM is a crucial component of the glomerular capillary wall and a significant factor in determining the size of selectivity of the glomerular filter.^27^ It is mainly a fibrous network composed of type IV collagen, laminin, and nitrogen. GBM thickening is an early morphological feature of DKD and can be used as a pathological hallmark for diagnosing DKD.^28^ Due to the positional relationship between podocytes and GEnCs, their abnormal cross talk is crucial in the pathogenesis of DKD **(**Figure 1 **)**. In summary, GEnCs, podocytes, PECs, and mesangial cells are interdependent in the glomerulus and jointly maintain the structure and function of the glomerulus.^29^

## 2 CROSS TALK BETWEEN GLOMERULUS CELLS

**FIGURE 2:**
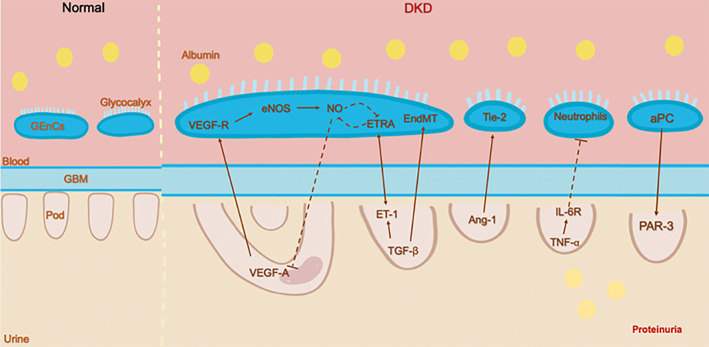
Summary of GEnCs‐podocytes cross‐talk signaling pathways in DKD. Activation and inhibition are indicated by arrows and dotted lines, respectively. Ang‐1: Angiopoietin‐1. aPC: Activated protein C. eNOS: Endothelial nitric oxide synthase. EndMT: mesenchymal transformation. ET‐1: Endothelin‐1. ETRA: ET receptor A. GBM: glomerular basement membrane. GEnCs: glomerular endothelial cells. IL‐6R: Interleukin‐6 Receptor. NO: nitric oxide. PAR‐3: Protease‐activated receptor‐3. Pod: podocyte. Tie‐2: Tyrosine‐protein kinase receptor 2. TGF‐β: Transforming growth factor β. TNF‐α: Tumor necrosis factor. VEGF‐A: Vascular Endothelial Growth Factor A. VEGF‐R: Vascular Endothelial Growth Factor Receptor.

Advances in the development of modern biotechnology have significantly increased our understanding of the communication between GEnCs, podocytes, and mesangial cells. These specific intercellular signaling pathways allow for the formation and maintenance of the GFB. In the progress of DKD, alterations in intraglomerular signaling may facilitate or aggravate the lesion. Figure 2 provides an illustration of the cross talk between GEnCs and podocyte pathways. While some of these interactions have been demonstrated, most remain unknown and deserve further investigation.

### 2.1 Cross talk between podocytes and GEnCs

#### 2.1.1 Vascular endothelial growth factor A signaling

The most well‐studied cross‐talk mechanism in the glomeruli is VEGF‐A/VEGFR (vascular endothelial growth factor A/vascular endothelial growth factor receptor) system.^30^ VEGF‐A is a significant regulator of blood vessel biology. The significant functions facilitate endothelium‐dependent vasodilatation and increased vascular permeability.^31^ VEGF‐A induces intracellular signaling by binding to one of two receptors: VEGFR‐1 (or Flt‐1) and VEGFR‐2 (or Flk‐1/KDR), and VEGFR‐2 mediates most of the biological effects of VEGF‐A under usual conditions.^32^ Existing studies have confirmed that the VEGF‐A/VEGFR signaling pathway is critical for glomerular development and renal homeostasis.^33^, ^34^

VEGF‐A is expressed predominantly by podocytes during glomerular development, while GEnCs and cortical and reno medullary interstitial fibroblasts express the VEGF receptors.^35^ In the glomerulus, the canonical VEGF signaling is the secretion of VEGF‐A by podocytes which then crosses the filtration barrier in opposition to urinary and acts by binding to VEGFR‐2 on the surface of GEnCs,^36^ forming the VEGF‐A/VEGFR axis. With the development of genetic models, we appreciate the importance of VEGF‐A imbalance in the diabetic setting. Using the Cre‐loxP technology, Vera Eremina et al. found that loss of both alleles of VEGF‐A in podocytes results in a marked reduction in endothelial cell migration into the developing glomeruli, which in turn leads to glomerular filtration over‐barrier failure.^37^ Conversely, excessive levels of VEGF‐A in endothelial cells can lead to cell swelling, loss of fenestrations, and even thrombotic microangiopathy.^38^ These results can demonstrate that podocytes and GEnCs interact with each other through the VEGF‐A/VEGFR signaling pathway. Numerous experimental studies have demonstrated that the expression of VEGF‐A and VEGFR‐2 increases in kidneys and urine in diabetic rats.^39^ However, when anti‐VEGF drugs are used in clinical tumor therapy, blocking VEGF signaling may lead to many common adverse vascular reactions, including hypertension and renal vascular injuries, such as proteinuria and thrombotic microangiopathy.^30^, ^40^, ^41^ Hence, the VEGF‐A/VEGF‐R signaling pathways play several critical roles in DKD and highlight the intricacies of an intraglomerular cross‐talk system. There are still many unanswered questions about this pathway, and it will be interesting to delineate how to regulate this signaling pathway beneficially in DKD.

#### 2.1.2 Endothelial nitric oxide synthase signaling

Endothelial nitric oxide synthase (eNOS) is expressed in endothelial cells and platelets, is one of the three NOS isoforms,^42^ and is involved in the production of nitric oxide (NO) in the vascular endothelium.^43^ NO has a variety of biological functions and affects a variety of actions in the vasculature, including dilating blood vessels, anti‐inflammatory, and antithrombotic.^44^ Clinical evidence suggests that impaired eNOS expression is related to the development of DKD^45^; glomerular eNOS expression was lower in patients with macroalbuminuria than in patients with microalbuminuria.^46^ NO produced by eNOS in the endothelial cells is an essential mediator for maintaining renal blood flow, glomerular filtration rate, and fluid homeostasis.^47^, ^48^ Several studies have shown that eNOS‐deficient diabetic mice are a successful model to simulate human DKD, enabling us to study the pathogenesis of progressive DKD;^49^; so studies on the role of eNOS in diabetic nephropathy have become more and more extensive in recent years. The intersection between VEGF‐A and NO pathways plays an essential role in the pathogenesis of DKD. Under normal conditions, VEGF‐A in podocytes induces the activation of eNOS in GEnCs, leading to NO production, which negatively regulates the amount of VEGF‐A. Appropriate VEGF‐A levels can be maintained in the glomerulus.^50^, ^51^ Interestingly, studies have shown that VEGF‐A and eNOS can be two independent events affecting the progression of diabetic nephropathy. Delma Veron found that induced VEGF‐A‐induced overexpression in eNOS‐deficient mice leads to significant proteinuria and glomerulosclerosis^52^; this confirms that eNOS can function independently. However, NO production is naturally reduced due to eNOS deficiency. NO increases glomerular albumin permeability, so it is unlikely that hyperalbuminuria in diabetic eNOS‐deficient mice is due to reduced NO production.^53^

Why diabetic eNOS‐deficient mice produce large amounts of proteinuria remains worthy of further investigation.

#### 2.1.3 Angiopoietin signaling

Angiopoietins (Ang) are a group of vascular growth factors that regulate vascular stability by controlling endothelial sprouting during angiogenesis.^54^, ^55^ Among the four angiopoietins (Ang 1‐4), Ang‐1 and Ang‐2 are two main subtypes.^56^, ^57^ In the glomerulus, Ang‐1 is mainly expressed in podocytes, and its primary function is through binding to tyrosine‐protein kinase receptor 2 (Tie‐2), which is expressed on the endothelial cell surface, promoting GEnC survival by limiting endothelial permeability.^58^ In recent years, many investigators have studied the role of Ang in DKD in genetically modified mice. They have demonstrated that the decrease in the Ang‐1/Ang‐2 ratio accelerated the development of DKD.^59^ Compared with nondiabetic animals, at the early stage of DKD, the expression level of Ang‐1 messenger RNA (mRNA) decreased, while the expression level of Ang‐2 mRNA did not change significantly.^59^ A study by Gnudi's team reported that podocyte‐specific induction of Ang‐1 overexpression leads to a significant reduction in proteinuria and prevents diabetes‐induced GEnC proliferation by increasing the Tie‐2 phosphorylation in adult diabetic mice.^60^ Conversely, mice induced with podocyte‐specific Ang‐2 overexpression showed markedly increased albuminuria and glomerular endothelial apoptosis, providing additional evidence for GEnC‐podocyte cross talk in the glomerulus.^61^

#### 2.1.4 Endothelin‐1 signaling

Endothelin‐1 (ET‐1), a significant member of the endothelin peptide family, functions in the kidney mainly through two isoforms of ET receptors highly expressed in the kidney, ETRA and ETRB.^62^ In the context of diabetes, ET‐1 is involved in vasoconstriction, mesangial proliferation, glomerulosclerosis, and fibrosis by activating ETRA.^63^ Animal experiments have documented that selective blockade of the ETRA reduces urinary albumin excretion, inflammatory marker production, and podocyte loss. However, in clinical trials, fluid retention and hepatotoxicity are common adverse reactions using ET receptor antagonists.^64^ GEnCs are coated with an endothelial surface layer (ESL), which consists of a membrane‐bound glycocalyx and a more loosely attached cell coat.^13^ In DKD experimental models, the loss of ESL is associated with albuminuria and precedes the effacement of podocyte FPs.^65^ Kerstin Ebefors et al. found that in the mouse models of primary podocytopathy or Adriamycin (AD) nephropathy, podocyte‐derived ET‐1 interacted with increased GEnCs ETRA expression resulting in the loss of ESL. This study also confirmed that increased heparanase (Hpse) (a degrading enzyme of the ESL) expression in GEnCs was in response to podocyte‐releasing factors and ET‐1.^66^ The content of ETRA is also closely related to NO. When the expression level of ETRA is low, the increase of NO can be induced, while when the content of ETRA is high, the production of NO is inhibited.^67^ These provide another compelling evidence for the podocyte pathologic cross talk with endothelial cells.

#### 2.1.5 Transforming growth factor β signaling

Transforming growth factor β (TGF‐β) is a pleiotropic cytokine that controls diverse cell types’ growth, development, inflammation, and function.^68^, ^69^ Massive evidence indicates that dysfunction of TGF‐β signaling is a key factor in DKD pathogenesis associated with podocyte loss, accumulation of ECM, and interstitial fibrosis.^70^, ^71^, ^72^, ^73^ TGF‐β mediates apoptosis and dedifferentiation of podocytes, even leading to podocyte abscission.^74^, ^75^ In a mouse model of glomerulosclerosis, Ilse Daehn's group determined that podocyte‐specific activation of TGF‐β signaling correlates with ET‐1 released by podocytes, which activate mitochondrial oxidative stress in adjacent GEnCs via paracrine ETRA and dysfunction.^76^ Studies have shown that TGF‐β induces mesenchymal transformation (EndMT) in GEnCs, leading to diabetic renal fibrosis.^51^, ^77^ Transcriptome analysis of GEnCs obtained from early diabetic mice revealed increased gene expression of leucine‐rich a‐2‐glycoprotein 1 (LRG1).^78^ Quan Hong demonstrated that ablation of LRG1 significantly reduced TGF‐β‐induced glomerular angiogenesis, podocyte loss, proteinuria, and glomerular lesions through decreased activation of activin receptor‐like kinase 1 (ALK1)‐Smad1/5/8.^79^ These results exemplify that the TGF‐β signaling pathway mediates podocyte damage and GEnC gene expression alterations in DKD.

#### 2.1.6 Interleukin‐6 signaling

Interleukin‐6 (IL‐6) is a well‐described multifunctional cytokine that regulates the immune and inflammatory response and affects insulin resistance, hematopoiesis, lipid metabolism, and organ development.^80^, ^81^ In the glomerulus, podocytes are the only cells that express the IL‐6 receptor.^82^ Sahithi J. Kuravi et al. co‐cultured podocytes and GEnCs and then stimulated them with tumor necrosis factor‐α (TNF‐α). Experimental results revealed that podocytes play a role in modulating neutrophil recruitment to GEnCs by releasing IL‐6. This result indicated that IL‐6 paracrine by podocytes has anti‐inflammatory effects.^83^ However, as the disease changes, so does the role of the IL‐6 pathway, which may lead to inflammation and even worsen glomerular disease.

#### 2.1.7 Activated protein C signaling

Activated protein C (aPC) is a plasma serine protease derived from its inactive precursor protein C (PC); the production of aPC requires the precise assembly of PC, endothelial PC receptor (EPCR) thrombin, and thrombomodulin on the surface of endothelial cells.^84^ Isermann et al. found that impaired aPC formation is associated with DKD, and the cytoprotective effect of aPC is mediated by preventing apoptosis in endothelial cells and podocytes.^85^ Thati Madhusudhan et al. further used genetic ablation of the protease‐activated receptor‐3 (PAR‐3) to demonstrate that aPC prevents lipopolysaccharides‐induced podocyte injury depending on protease‐activated receptors conveying the signal in podocytes.^86^ These suggest that aPC regulates DKD through the cross talk between vascular areas, endothelial cells, and podocytes.

#### 2.1.8 Krüppel‐like factor 2 signaling

Krüppel‐like factor 2 (KLF2) is a shear stress‐inducible transcription factor whose primary role is to protect endothelial cells and maintain vascular integrity.^87^ Studies have shown that KLF2 expression is downregulated in GEnCs of patients with DKD, and its lack accelerates disease progression. An interesting study showed that podocyte loss was also increased in diabetic KLF2^EC/+^ mice compared to diabetic wild‐type mice, suggesting that KLF2 is involved in GEnCs‐podocytes cross talk and that targeting KLF2 may be a novel strategy to prevent DKD progression.^88^

### 2.2 Cross talk between GEnCs and mesangial cells

#### 2.2.1 Integrin αvβ8

Integrins function as cell membrane receptors for ECM components to maintain tissue integrity and mediate cell adhesion, migration, and proliferation with neighboring matrix.^89^ The changes in glomerular integrin expression have been demonstrated in both patient and animal models of DKD.^90^ Integrins are noncovalently bound heterodimers consisting of α and β subunits. In invertebrates, the 18α and 8β subunits can be combined into 24 different receptors, each with different binding properties and tissue distribution.^28^ Among mesangial cells, αvβ8 is the most abundantly expressed in the integrin family, and its primary ligand is TGF‐β.^91^ Henrik Dimke et al. have constructed integrin αVβ8‐deficient mouse models with two different genetic backgrounds, resulting in glomerular dysfunction, endothelial apoptosis, and albuminuria in mice, and these effects were ascribed to regulating the bioavailability of TGF‐β.^92^ This result demonstrated that reciprocal communication involving mesangial cell‐derived molecules could affect GEnCs.

#### 2.2.2 PDGF‐B/PDGF‐Rβ signaling

Platelet‐derived growth factor‐B (PDGF‐B) is a high‐affinity ligand for the tyrosine kinase receptors PDGF‐Rα and PDGF‐Rβ.^93^ In the glomerulus, GEnCs express PDGF‐B, and its receptor PDGF‐Rβ resides on mesangial cells.^94^

Using in situ hybridization, Lindahl et al. demonstrated that PDGF‐B acted paracrine during kidney development.^95^ Mice deficient in GEnC PDGF‐B showed significantly reduced mesangial cells. Recent findings have highlighted that tissue hypoxia is one of the central pathways of DKD.^96^, ^97^ According to a study, hypoxia can increase PDGF‐B expression in GEnCs, and eliminating the exaggerated response of hypoxic mesangial cells to the PDGF‐B pathway may be a strategy for treating DKD.^98^

### 2.3 Cross talk between podocytes and mesangial cells

Unlike extensive studies on other cells, the direct evidence for communication between mesangial cells and podocytes has been scarce. Through single‐nucleus RNA sequencing, differentially expressed ligand receptor intercellular signaling pathways and all possible ligand receptor signaling pathways have been identified in podocytes and mesangial cells.^99^ Researchers have found that podocyte injury often leads to mesangial cell proliferation, while mesangial cell damage results in FP fusion, but the specific mechanism remains unclear.^100^

### 2.4 New mediators of glomerulus cell cross talk

#### 2.4.1 Role of microRNAs in glomerulus cells cross talk

MicroRNAs (miRNAs) are a class of small noncoding RNAs with 21–25 nucleotides, which correspond to complementary sequences in the 3'‐untranslated regions (3'UTR) of target mRNAs or open reading frames within the target gene.^101^ In the past decades, they have become the focus of DKD research, emerging as biomarkers, regulators, and potential targets of future therapies.^102^, ^103^ Recently, a paracrine role of miRNAs in the communication between glomerulus cells has been reported. Janina Müller‐Deile and colleagues demonstrated that after stimulation with TGF‐β, the expression of miRNA‐143 in podocytes is increased and resulted in the down‐regulation of glycocalyx proteins in podocytes and GEnCs and structural impairments of the GFB. These results may illustrate that miRNA‐143 is a mediator for glomerular cross talk by affecting the glomerular glycocalyx.^104^ There are still relatively few studies on miRNAs in glomerulus cell cross talk, and it is of great value to further explore the role of miRNAs in DKD.

#### 2.4.2 Extracellular vesicles as new mediators of cell‐cell cross talk in DKD

Extracellular vesicles (EVs) are endogenously produced membrane‐bound vesicles. All cells release EVs as part of their normal physiology and during acquired abnormalities.^105^ Depending on their size and biogenesis and release mechanisms, EVs can be broadly classified into two major subgroups, plasma membrane‐derived ectosomes (microvesicles [MVs] and microparticles) and endosome‐origin exosomes.^106^ MVs are 50 nm to 1 mm in diameter, whereas exosomes are small vesicles in the 50–150‐nm range.^107^ Initially, exosomes were thought to be exocytic vesicles only to be used to shed some intracellular and membrane components out of the cells.^108^ In recent years, exosomes have been proven to be a novel biomarker that plays a crucial role in maintaining cellular homeostasis by delivering proteins, mRNAs, and microRNAs as nanocarriers to communicate with neighboring or distant cells.^109^

With the development of second‐generation sequencing technology, the research on DKD exosomes has progressed rapidly and is expected to become a new target for the clinical treatment of DKD.^110^ Through cell–cell cross talk, EVs secreted by damaged kidney cells can be transferred to other normal kidney cells. For instance, compared with normal glucose (NG)‐treated GEnCs, high glucose (HG)‐treated GEnCs secreted increased levels of TGF‐β1 in exosomes and promoted mesangial cell proliferation, α‐smooth muscle actin expression, and ECM protein excess.^111^ Xiaoming Wu et al. showed that the TGF‐β1 mRNA in exosomes from HG‐treated GEnCs can also mediate the epithelial‐mesenchymal transition and dysfunction of podocytes.^4^ In addition to transmitting damage signals, exosomes also have protective effects. Olivier G et al. showed that exosomes produced from hypoxic endothelial cells promoted endothelial cell repair by upregulating lysine oxidase‐like 2 and increasing collagen cross‐linking activity.^112^ Recent finding demonstrated that miRNAs can be packaged into exosomes and secreted from cells.^113^ A study by N. Hill et al. showed that by stimulating GEnCs with glucose or puromycin aminonucleoside, exosomes secreted by GEnCs increased the content of miRNA‐200c‐3p in podocytes and reduced VEGF production, which may lead to kidney disease.^114^ Hong Su et al. found that under high‐glucose conditions, microRNA‐221 in exosomes secreted by podocytes leads to dedifferentiation of proximal tubule cells through the Wnt/β‐catenin pathway.^115^ These findings suggest that EVs can be potential mediators for glomerulus cell cross talk in DKD and may be further explored as diagnostic markers and therapeutic targets for DKD.

## 3 CONCLUSIONS AND PERSPECTIVES

Besides the significant mediators, such as VEGF‐A, Ang, and eNOS, researchers have also discovered miRNAs, exosomes, and other new mediators that participate in glomerular cell‐cell cross talk. Under normal physiological conditions, this communication system is adaptive for glomerular function, but during DKD, it becomes maladaptive and accelerates the onset of DKD. Despite the research base and potential, the study of cross talk in the glomerulus is in its infancy, and there are still many questions to be asked and answered. The pathogenesis of diabetic nephropathy is complex, and the cells in the glomerulus are in different environments and dimensions, so cell‐specific transmission remains a great challenge, requiring multidisciplinary cooperation. For example, the study by Tan et al. used three‐dimensional (3D) multiscale model simulation of podocyte injury and repair under various cytokine disturbances in healthy and diabetic conditions.^116^ In addition to 3D technology, the continuous development of new technologies such as RNA sequencing and renal organoid will provide more possibilities to discover the interactions between glomerular cells and lead to new directions for the future treatment of DKD.

## DISCLOSURE

The authors declare that there is no conflict of interest regarding the publication of this paper.

## AUTHOR CONTRIBUTIONS

Youhua XU provided the ideas for this paper, and Ruixue DONG conducted the literature search and the first draft. Youhua XU reviewed and edited the manuscript before submission. All authors read and approve the final draft.
